# Multi-view registration of unordered range scans by fast correspondence propagation of multi-scale descriptors

**DOI:** 10.1371/journal.pone.0203139

**Published:** 2018-09-10

**Authors:** Siyu Xu, Jihua Zhu, Zutao Jiang, Zhiyang Lin, Jian Lu, Zhongyu Li

**Affiliations:** 1 School of Software Engineering, Xi’an Jiaotong University, Xi’an, China; 2 School of Electronic and Information, Xi’an Polytechnic University, Xi’an, China; 3 University of North Carolina at Charlotte, Charlotte, North Carolina, United States of America; Technische Universiteit Delft, NETHERLANDS

## Abstract

This paper proposes a global approach for the multi-view registration of unordered range scans. Our method starts with the pair-wise registration, where multi-scale descriptor is selected for feature point and the propagation of feature correspondence is accordingly accelerated. Subsequently, we design an effective rule to judge the reliability of these pair-wise registration results. According to the judgment of reliability, we propose a model fusion method, which can utilize reliable results of pair-wise registration to augment the model shape. Finally, multi-view registration can be achieved by operating the pair-wise registration, reliability judgment, and model fusion alternately. The proposed approach can be applied to scene reconstruction and robot mapping. Experimental results conducted on public datasets show that the proposed approach can automatically achieve multi-view registration of unordered range scans. Compared with other related approaches, the proposed approach has superior performances in accuracy and effectiveness.

## Introduction

Registration of range scans is a fundamental task, which has attracted great attention in many research fields, such as robot mapping [1–3], computer vision [4] and computer graphics [5,6]. Given a global reference frame, the goal of registration is to find optimal transformation(s) for one or more scans so as to transfer all scans into one unified frame. According to the number of scans to be registered, this problem can be divided into pair-wise registration and multi-view registration.

For pair-wise registration, Besl et al. [7] proposed the iterative closest point (ICP) algorithm, which is one of the most popular registration approaches. Although this approach can achieve the pair-wise registration with good efficiency, it is unable to get desired results for the registration of partially overlapping range scans. Besides, it is a local convergent approach. To obtain the desired results, good initial parameters should be provided to the ICP algorithm. For registration of partially overlapping scans, Chetverikov et al. [8] proposed the trimmed ICP (TrICP) algorithm, which introduces the overlap parameter to determine overlapping parts so as to estimate accurate rigid transformation. Further, Phillips et al. [9] improved the performance of original TrICP algorithm with high efficiency. To address the local convergence, Genetic algorithm (GA) [10,11] and particle filtering [12] were applied to global registration of scan pairs. To guarantee global optimality, reasonable initializations are still required; otherwise spaces of parameters are too large for heuristic search. What’s more, these global optimization approaches require high computation complexity. Subsequently, Yang et al. [13] proposed the first globally optimal algorithm, named Go-ICP. This approach is based on a branch-and-bound (BnB) scheme that searches the entire 3D motion space and able to produce reliable registration results regardless of the initialization. Besides, many feature matching based approaches have been proposed for the pair-wise registration [14–16]. Among these approaches, one of the most efficient and robust approaches is multi-scale descriptors with correspondence propagation [16], which can be further improved in efficiency.

Although these above-mentioned approaches may obtain good results for pair-wise registration, most of them cannot deal with multi-view registration. Given a set of range scans, the task of multi-view registration is to find optimal transformations of each scan to the reference scan. Compared with pair-wise registration, multi-view registration is somewhat more difficult due to many transformations required to be estimated. In recent years, many solutions have been proposed to solve this problem. Among these solutions, the sequential registration approach is the simplest one [17]. For multi-view registration, it alternately aligns and integrates two scans until all scans are integrated into one model. Although this approach is simple, it suffers from the error accumulation problem. To address this issue, Zhu et al. [18] proposed the coarse-to-fine registration approach, which sequentially aligns each scan to the model integrated by all other aligned scans. By traversing all scans, all transformations are sequentially refined by the pair-wise registration. Meanwhile, Govindu et al. [19] proposed a multi-view registration approach based on motion averaging algorithm, which takes a set of available relative motions (pair-wise registration results) as input to simultaneously estimate all transformations for multi-view registration. Based on this work, Guo et al. [20] proposed the weighted motion averaging algorithm for multi-view registration. This approach can improve the robustness and accuracy of multi-view registration by paying more attention to reliable pair-wise registration results. Recently, Arrigoni et al. [21] proposed a multi-view registration approach based on the low-rank and sparse (LRS) matrix decomposition. It stacks all available relative motions into a large matrix and replaces the unavailable ones with zeros, then utilizes the LRS decomposition to recover global motions for multi-view registration. Moreover, Georgios et al. [22] cast the multi-view registration problem into the framework of clustering. For clustering, it utilizes the Expectation-Maximization (EM) algorithm to simultaneously estimate Gaussian Mixture Model (GMM) and all transformations for multi-view registration.

Although these above discussed approaches may obtain desired results for multi-view registration, they should be provided with fine initial registration parameters. Otherwise, it is difficult to obtain desired registration results. For the global multi-view registration, Daniel et al. [23] proposed the approach for fully automatic registration of multiple range scans. It applied the pair-wise registration on all scan pairs involved in multi-view registration and then tested these results for surface consistency. Accordingly, reliable results of pair-wise registration can be checked and selected for the estimation of multi-view registration. Since there are many scan pairs involved in multi-view registration, the computation complexity of this approach is very high. Subsequently, Guo et al. [24] proposed a feature match based approach for global multi-view registration. This approach is also based on the pair-wise registration, which is more efficient than previous approaches. However, it sometimes cannot find the minimum number of good feature matches for pair-wise registration, which leads to the failure of multi-view registration. Recently, Zhu et al. [25] proposed the global multi-view registration approach based on the construction of spanning tree. It designs the dual criterion to judge the reliability of pair-wise registration results. By viewing the first scan as the root node, all other scans can be added by the breadth-first search with the reliable results of the pair-wise registration. As most scan pairs contain low overlap percentages, it is also required to align and check many scan pairs to construct a full spanning tree. Therefore, its computation complexity is also high.

Taking these above problems into account, this paper proposes a global approach for the multi-view registration of unordered range scans. The contribution of this paper can be delivered as follows: 1) It accelerates correspondence propagation of multi-scale descriptor for pair-wise registration; 2) It designs an effective rule to judge the reliability of pair-wise registration; 3) It proposes the model fusion to merge scan pairs, which can be successfully aligned. To demonstrate its effectiveness, the proposed approach is tested on public available data sets and compared with two other related registration approaches.

The reminder of this paper is organized as follows. We briefly review related work of pair-wise registration approach in Section 2. In Section 3, we present details of the proposed multi-view registration approach. All experimental results are displayed in Section 4. Section 5 demonstrates applications of the proposed approach in scene reconstruction and robot mapping. Finally, conclusion and future works are discussed in Section 6.

## Pair-wise registration

This section briefly reviews TrICP algorithm and the correspondence propagation of multi-scale descriptors for pair-wise registration.

### TrICP algorithm

Suppose there are two partially overlapping scans in ${\mathbb{R}}^{3}$, the data shape $P≜{\left\{p_{i}\right\}}_{i=1}^{N_{p}}$ and the model shape $Q≜{\left\{q_{j}\right\}}_{j=1}^{N_{q}}$ $\left(N_{p},N_{q}\in \mathbb{N}\right)$, where *ξ* denotes the overlap percentage and *P*_*ξ*_ indicates the overlapping part of *P* to *Q*. Accordingly, the overlap percentage can be calculated as $\xi =N_{p}^{'}/N_{p}$, where $N_{p}^{'}$ denotes the number of point included in the subset *P*_*ξ*_. Subsequently, it is convenient to define trimmed mean square error (TMSE) as:

$$e(\xi ,R,t)=\frac{1}{N_{p}^{'}}\sum_{{\vec{p}}_{i}\in P_{\xi }}\left({\left\|Rp_{i}+t-q_{c(i)}\right\|}_{2}^{2}\right)$$ (1)

where **R** is the 3×3 rotation matrix, **t** is the 3×1 translation vector and (**p**_*i*_,**q**_*c*(*i*)_) denotes a pair of point correspondence. According to [8], the task of pair-wise registration is to find an optimal rigid transformation (**R**,**t**), which can be obtained by minimizing the following objective function:

$$\psi (\xi ,R,t)=\frac{e(\xi ,R,t)}{{\left(\xi \right)}^{1+\lambda }}$$ (2)

where *λ* is a preset parameter (*λ* = 2 in this paper).

Currently, Eq (2) has been solved by the TrICP algorithm, which achieves pair-wise registration by iterations. Given initial parameters $\left(R_{0},{\vec{t}}_{0}\right)$, three steps are included in each iteration:

1. Establish point correspondences between two range scans:
  $$c_{k}(i)=\underset{j\in \left\{1,2,\ldots ,N_{q}\right\}}{\arg\;\min}{\left\|R_{k-1}p_{i}+t_{k-1}-q_{j}\right\|}_{2},i=1,2,\ldots ,N_{p}$$ (3)
2. Update the overlapping percentage and its corresponding subset:
  $$(\xi_{k},P_{\xi_{k}})=\underset{\xi_{\min}<\xi \leq 1}{\arg\;\min}\;\sum_{{\vec{p}}_{i}\in P_{\xi }}{\|R_{k-1}p_{i}+t_{k-1}-q_{c_{k}(i)}\|}_{2}^{2}/\left(\left|P_{\xi }\right|\xi^{1+\lambda }\right)$$ (4)
3. Calculate the new transformation:
  $$(R_{k},t_{k})=\underset{R,t}{\arg\;\min}\sum_{{\vec{p}}_{i}\in P_{\xi_{k}}}{\left\|Rp_{i}+\vec{t}-q_{c_{k}(i)}\right\|}_{2}^{2}$$ (5)

A rigid transformation $\left(R,\vec{t}\right)$ will be obtained by repeating steps (1)-(3) until the iteration *k* reaches the maximum number *K* or |*ψ*_k_-*ψ*_k-1_|<*ε*, where *ε* denotes a given small positive number. Similar to the original ICP algorithm, the TrICP algorithm is also local convergent. To obtain desired results, fine initial parameters should be provided.

### Correspondence propagation of multiscale descriptors

**Fig 1:**
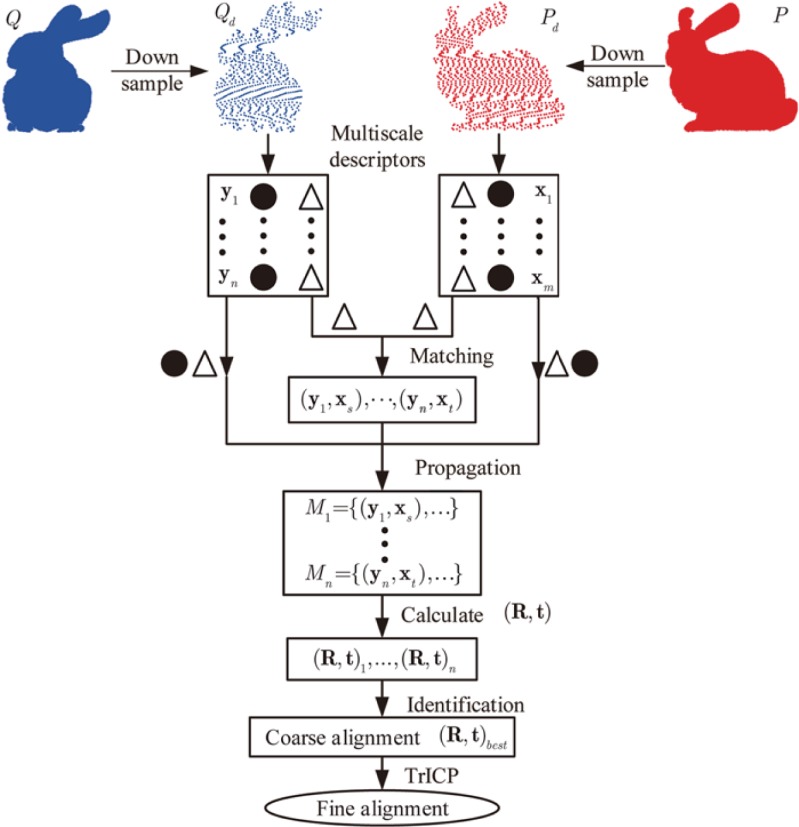
Flow chart of the pair-wise registration approach based on correspondence propagation of multi-scale descriptors.

For the pair-wise registration, multi-scale descriptors have been proposed in [16]. Given a point, *L* different support radii are considered. Within each support radius, all included range points are used to compute the variance matrix **C**_*l*_. Based on these *L* circles and variance matrix, it is convenient to compute multi-scale descriptors (**N**,**D**), where **N** denotes the normal vector and **D** indicates the eigenvalue based vector. Accordingly, the pair-wise registration can be solved by the effective approach displayed in Fig 1.

As shown in Fig 1, after the calculation of multi-scale descriptors, the correspondence of each point from **y**_1_ to **y**_*n*_ is established by performing the nearest neighbor (NN) search on the eigenvalue based vector **D**. For correspondence propagation, each correspondence of these points can be viewed as a seed match. Then, the propagation session exploits both **D** and **N** to propagate each seed match (e.g., (**y**_1_,**x**_*s*_)) into a set of matches (e.g., *M*_1_ = {(**y**_1_,**x**_*s*_),…}). For each match set, the RANSAC algorithm should be used to find the maximum consensus set so as to calculate one optimal rigid transformation. Based on down sampled scans *Q*_*d*_ and *P*_*d*_, a quality function formulated from distances error is defined to identify the best one from all obtained rigid transformations. Finally, the TrICP algorithm refines the results of pair-wise registration by taking the best transformation (**R**,**t**)_*best*_ as its input.

According to [16], the time complexity of each propagation operation is about *O*((*m*+*n*)log*n*), where *n* and *m* denotes the number of points included in the down sampled scans *Q*_*d*_ and *P*_*d*_, respectively. As the required number of propagation is *n*, the total complexity of correspondence propagation is *O*(*n*(*m*+*n*)log*n*). Although this approach is robust for global pair-wise registration, the operation of correspondence propagation is too time-consuming.

## Global registration of multi-view scans

**Fig 2:**
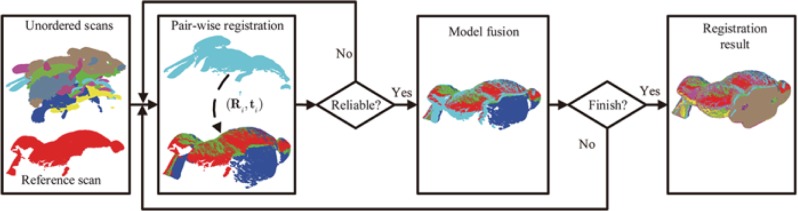
The flowchart of the proposed approach for multi-view registration, which operates pair-wise registration, the judgment of pair-wise registration result and model fusion alternately until all range scans are integrated into one model.

For multi-view registration of unordered scans, a global approach is presented in Fig 2. Given a set of unordered scans, the frame of reference, without loss of generality, can be attached to the first scan. As shown in Fig 2, the proposed approach includes three main operations; there are pair-wise registration, reliability judgment of pair-wise registration and model fusion. By alternately implementing these three operations, the proposed approach can achieve multi-view registration with good performance. Subsequently, we will present more details of these three operations and the implementation of this approach.

### Pair-wise registration by fast correspondence propagation

For the unordered range scans, there is no prior information about initial parameters in the stage of global registration. Therefore, Lei et al. [16] proposed the multi-scale descriptors with correspondence propagation for global pair-wise registration. As shown in Fig 1, this approach requires to do several operations to achieve pair-wise registration. Among these operations, correspondence propagation is the most important and time-consuming one.

**Fig 3:**
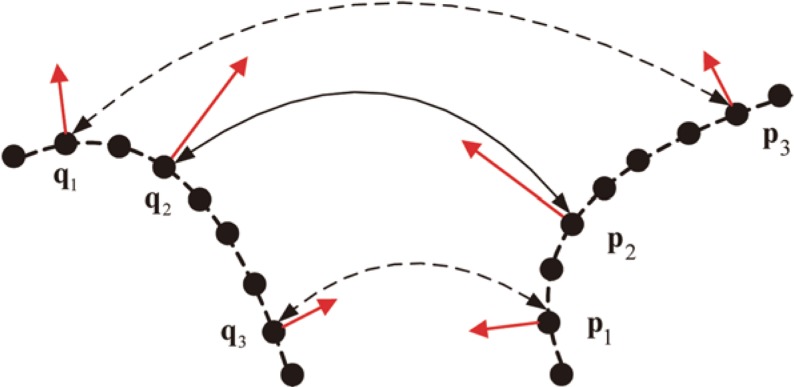
Demonstration of different seed matches, one solid line with bi-directional arrow denotes a reliable seed match, each dotted line with bi-directional arrow denotes an unreliable seed match, the direction and length of red line with arrow denote the normal vector and the eigenvalue based vector, respectively.

According to [16], multi-scale descriptors consist of the normal vector **N** and the eigenvalue based vector **D**, where **D** is rotation invariant. Hence, seed matches are established by performing the NN search on **D**. But these seed matches may be unreliable due to the low dimension of **D**. To obtain reliable point correspondences, it is necessary to apply correspondence propagation. However, it is not required to apply correspondence propagation on each seed match. Because once the propagation starts from one reliable seed match, the exploitation of **N** and **D** will certainly propagate it into a set of good matches. Accordingly, some reliable seed matches are enough for the correspondence propagation of multi-scale descriptors. The problem is how to find reliable seed matches. Fig 3 illustrates both reliable and unreliable seed matches. Actually, ${\left(p_{i},q_{i}\right)}_{i=1}^{3}$ denote three reliable matches. But (**p**_1_,**q**_3_) and (**p**_3_,**q**_1_) may be mismatched due to noises or other reasons. Therefore, one seed match (**p**_1_,**q**_3_) or (**p**_3_,**q**_1_) with small distance of the eigenvalue based vector may not be reliable. While, the reliable seed match (**p**_2_,**q**_2_) must contain small distance. To this end, it is better to sort all established seed matches in ascending order by their distances of the eigenvalue based vector, then the correspondence propagation is only applied to top *δ* × 100% sorted seed matches. In this case, the total time complexity of correspondence propagation can be seriously reduced so as to result in more efficient pair-wise registration. Based on the above discussions, an efficient pair-wise registration algorithm is summarized in Algorithm 1.

**Algorithm 1. Efficient pair-wise registration algorithm**

**Input**: Scan pair *P* and *Q*

    Get *P*_*d*_ and *Q*_*d*_ by down sample;

    Calculate descriptors (**N**,**D**) for each point in *P*_*d*_ and *Q*_*d*_;

    Establish seed matches by performing the NN search on **D**;

    Sort all seed matches in the ascending order by their distances of **D**;

    For *i* = 1:*δn*

        Propagate the *i*th seed match into a set of matches *M*_*i*_ ={(**y**_*i*_,**x**_*c*(*i*)_),…};

        Find maximum consensus set in *M*_*i*_ by RANSAC;

        **If** (|*M*_*i*_|>10)

            Use *M*_*i*_ to calculate (**R**,**t**)_*i*_;

        **End**

    **End**

    Find the best alignment (**R**,**t**)_*best*_ by the quality function;

    Utilize TrICP to refine (**R**,**t**)_*best*_ and obtain (**R**,**t**).

**Output:** Pair-wise registration results (**R**,**t**)

In this algorithm, the required number of correspondence propagation is *δn*. As the propagation of each seed match is same to that of the method proposed in [16], the total complexity of correspondence propagation is reasonably reduced to *O*(*δn*(*m*+*n*)log*n*). Obviously, small value of *δ* will lead to low time complexity. Since the seed match with small distance may not be reliable, the value of *δ* should not be set too small. Otherwise, there may be no way to apply correspondence propagation to reliable seed match, which will lead to unexpected pair-wise registration. The choosing of parameter *δ* will be discussed in the experiment part.

### Reliability judgment of pair-wise registration

For these scan pairs involved in multi-view registration, some of them may contain high overlapping percentages, other may have low percentages or even no overlapping areas. Given a pair of scans, one rigid transformation will be estimated by the proposed pair-wise registration algorithm. For the scan pair with high overlapping percentage, the estimated transformation is reliable. However, it is unreliable for these scan pairs without high overlap percentages. Generally, it is difficult to predict or estimate the overlapping percentage for each scan pair, so the pair-wise registration may be applied to scan pair without high overlapping percentage and obtain unreliable transformation. As shown in Fig 2, only reliable results can be utilized for the model fusion. Therefore, it is required to judge the reliability of pair-wise registration.

**Fig 4:**
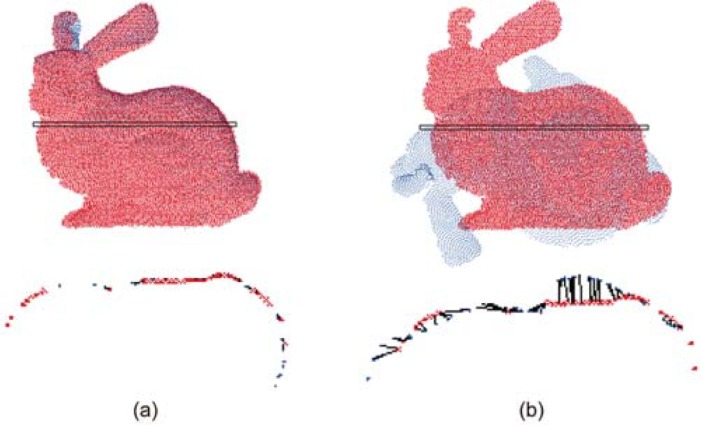
The illustration of TMSE for pair-wise registration of two scan pairs. In the 2nd row, each black line denotes the distance of one point pair located in the overlapping area. (a) Reliable registration. (b) Unreliable registration.

As the TrICP algorithm is finally utilized to refine the result of pair-wise registration, it also determines overlapping areas and provides TMSE for each scan pair. Fig 4 displays pair-wise registration results of two scan pairs, where one scan pair gets reliable registration due to its high overlap percentage and the other one gets unreliable registration due to its low overlap percentage or other reasons. As shown in Fig 4, the TrICP algorithm determines suitable overlapping areas with small TMSE for reliable registration and finds false overlapping areas with very large TMSE for the unreliable registration. Therefore, TMSE is related to the reliability of pair-wise registration.

**Fig 5:**
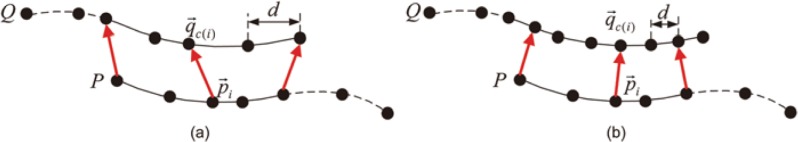
TMSEs under different point resolutions of model shape. (a) Low resolution results in large trimmed MSE. (b) High resolution reduces the trimmed MSE.

For the same registration result, the TMSE is affected by the point resolution of model shape. Fig 5 demonstrates the same pair-wise registrations under different point resolutions of model shape. As shown in Fig 5, high resolution reduces the TMSE and low resolution increases the TMSE. Therefore, we can judge the pair-wise registration is reliable if the TMSE satisfies the following condition:

$$TMSE\leq \alpha d_{Q}$$ (6)

where *d*_*Q*_ denotes the point resolution of model shape. Otherwise, the pair-wise registration is judged to be unreliable. To achieve multi-view registration, the pair-wise registration should be implemented many times in the proposed approach. During multi-view registration, it is easy to collect TMSEs from reliable registrations, which can be further utilized to judge the reliability of posterior registration results. Therefore, it is better to revise Eq (6) as follows:

$$TMSE\leq \max(\alpha d_{Q},\beta m_{TMSE})$$ (7)

where *m*_*TMSE*_ indicates the mean TMSE of reliable registrations. Before the first reliable registration has been checked, *m*_*TMSE*_ is set as *m*_*TMSE*_ = 0. After the check of one new reliable registration, it can be further updated. Here, *a* and *β* are two free parameters, which will also be discussed and determined in the Section of experiment.

### Model fusion

**Fig 6:**
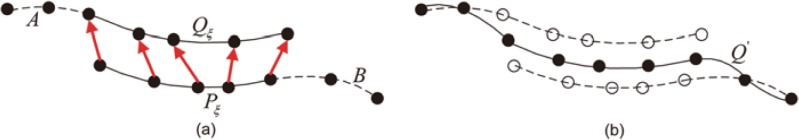
Illustration of model fusion. (a) The point pairs located in the overlapping area, where each pair of points is connected by one line with arrow. (2) Model and data shape is merged into one shape, where each point pair is replaced by its intermediate point in the overlapping area.

By the proposed judgment, it is easy to judge whether the pair-wise registration is reliable or not. For reliable registration, data shape should be transformed and merged into the model shape. The simplest method is to directly add points of transformed data shape into the model shape. However, this rude fusion will result in redundant range points with multi-scale descriptors, which can reduce the efficiency of subsequent registration. Hence, model fusion should be carefully designed. Accordingly, we propose the model fusion illustrated in Fig 6.

Based on the estimated rigid transformation (**R**,**t**), the data shape is first transformed into the reference frame of model shape as follows:

$$P^{'}≜{\left\{Rp_{i}+t\right\}}_{i=1}^{N_{p}}.$$ (8)

Then both model shape and transformed data shape are divided into two independent parts:

$$Q=Q_{\xi }\cup A,\;P^{'}=P_{\xi }^{'}\cup B,$$ (9)

where *Q*_*ξ*_ and *P*_*ξ*_ denotes overlapping parts of these two shapes, *A* and *B* indicates non-overlapping parts of them. Accordingly, the fused model shape is composed of raw model shape and transformed data shape as follows:

$$Q^{'}=A\cup F\cup B$$ (10)

where $F={\left\{f_{i}\right\}}_{i=1}^{N_{f}}$, *N*_*f*_ = *ξN*_*p*_, and

$$f_{i}=\frac{(Rp_{i}+t)+q_{c(i)}}{2}.$$ (11)

Similar to these two raw shapes, the down sampled data shape *P*_*d*_ should be correspondingly transformed and merged into the down sampled model shape *Q*_*d*_. And they can be merged in the same way as these raw shapes. Besides, multi-scale descriptors should also be merged. As eigenvalue based vectors **D** are rotation-invariant, they can be merged as follows:

$$D_{Q^{'},i}=\frac{\left(D_{P,i}+D_{Q,c(i)}\right)}{2},$$ (12)

where **D**_*P*,*i*_ and **D**_*Q*,*c*(*i*)_ denote eigenvalue based vectors of two matched points. While, normal vectors **N** are rotation-invariant, it can be merged as follows:

$$N_{Q^{'},i}=\frac{RN_{P,i}+N_{Q,c(i)}}{2}$$ (13)

where **N**_*P*,*i*_ and **N**_*Q*,*c*(*i*)_ indicate normal vectors of two matched points. In this way, the model shape is properly fused with corresponding multi-scale descriptors, so there is no need to recalculate multi-scale descriptors for the fused model shape.

Compared with rude fusion, the proposed fusion method can delete redundant range points with multi-scale descriptors.

### Implementation

The overall process of the proposed approach can be summarized in **Algorithm** 2.

**Algorithm 2: Multi-view registration of unordered range scans**

**Input**: A scan set with *N* unordered scans ${\left\{P_{i}\right\}}_{i=1}^{N}$ View the 1st scan as *Q* and delete it from scan set;

    Set *m*_*TMSE*_ = 0, *N*' = 0

    **Do**

  *N*' = 0;

  **For** i = 1:*N*

        View *P*_*i*_ as *P*, estimate (**R**_*i*_,**t**_*i*_) and *TMSE*_*i*_ by **Algorithm 1**;

        Use Eq (7) to judge the reliability of (**R**_*i*_,**t**_*i*_);

        **If** ((**R**_*i*_,**t**_*i*_) is reliable)

            *N*' = *N*' + 1;

            Update *Q* by the **Sec. 3.3**;

            Delete *P*_*i*_ from scan set;

            Recalculate *m*_*TMSE*_ and *d*_*Q*_;

    Record (**R**_*i*_,**t**_*i*_);

        **End**

    **End**

  *N* = (*N* − *N*');;

  **While** (*N* > 0)

**Output:** Multi-view registration results ${\left\{(R_{i},t_{i})\right\}}_{i=1}^{N}$

Given a set of unordered range scans, the proposed approach can automatically achieve global multi-view registration without any prior information.

## Results and discussion

To validate the performance of our proposed method, quantitative experiments were conducted on four data sets taken from the Stanford 3D Scanning Repository [26], which provides multi-view scans with ground truth of rigid transformations ${\left\{(R_{i,g},t_{i,g})\right\}}_{i=1}^{N}$. For calculating multi-scale descriptors, raw range points of each scan were down sampled with the sampling frequency set to be about 100. Besides, raw range points of each scan were down sampled with the sampling frequency set to be 10 for the pair-wise registration. For quantitative comparison, rotation error and translation error are defined as $e_{R}=\frac{1}{N}\sum_{i=1}^{N}{\left\|R_{i,m}-R_{i,g}\right\|}_{F}$ and $e_{t}=\frac{1}{N}\sum_{i=1}^{N}{\left\|t_{i,m}-t_{i,g}\right\|}_{2}$, where ${\left\{(R_{i,m},t_{i,m})\right\}}_{i=1}^{N}$ denotes rigid transformations estimated by multi-view registration approach. In all multi-view registration approaches, the NN search method based on *k-d* tree [27] was utilized to establish point correspondences. All multi-view registration approaches were implemented in Matlab on a desktop with four-core 3.6GHz processor and 8GB of memory.

### Parameter initialization

**Fig 7:**
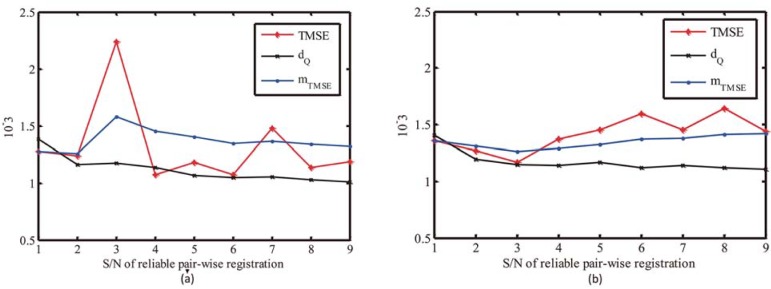
Illustration of *TMSE*, *d*_*Q*_,and *m*_*TMSE*_ in each reliable pair-wise registration. (a) Bunny. (b) Armadillo.

In the proposed approach, three free parameters require to be chosen. To choose *α* and *β*, we can fix the setting of *δ* = 1.0 and then test the proposed approach on Stanford Bunny and Armadillo. For each reliable pair-wise registration of multi-view registration, the value of trimmed mean square error *TMSE*, the point resolution of model shape *d*_*Q*_, and the mean TMSE of reliable registrations *m*_*TMSE*_ are recorded in Fig 7. As shown in Fig 7, the values of *TMSE* show significant change in each reliable pair-wise registration. Usually, *TMSE* and *m*_*TMSE*_ are slightly larger than *d*_*Q*_. Therefore, *α* and *β* should be assigned with large values so as to pick out all reliable pair-wise registration. However, large *α* and *β* will introduce unreliable pair-wise registration, which will lead to the failure of multi-view registration. Actually, unreliable pair-wise registration is caused by the low overlap percentage of data and model shapes. With model fusion, low overlap percentage will become high. That means unreliable pair-wise registration will turn to be reliable after model fusion. Considering these factors, *α* and *β* can be assigned with small values so as to avoid the involvement of unreliable pair-wise registrations. In following experiments, we set *α* = 2 and *β* = 1.5, which can result in robust multi-view registration.

After choosing *α* and *β*, we assign different values to *δ* and then test the proposed approach on Stanford Bunny and Armadillo. For each value of *δ*, the required number of pair-wise registration, run time and registration error of the proposed approach are recorded in Table 1. As shown in Table 1, within reasonable range, small value of *δ* will result in efficient registration without loss of registration accuracy. However, too small value of *δ* will lead to the failure of multi-view registration. For some scan pairs without high overlap percentages, there are small numbers of reliable seed matches. Under small value of *δ*, these reliable seed matches are all outside the top *δ* × 100%, which can lead to the failure of correspondence propagation and further result in registration failure. Accordingly, it is unreasonable to assign *δ* with too small value. By considering both robustness and efficiency, we set *δ* = 0.3 with enough tolerance. During experiments, we find this setting can lead to robust and efficient registration of multi-view scans.

**Table 1:** Results of the proposed approach with different values of *δ* on the Bunny and Armadillo datasets.

	Bunny				Armadillo			
	Pair	Time(min.)	*e*_R_	e_t_	Pair	Time(min.)	*e*_R_	e_t_
*δ* = 0.1	+∞	+∞	/	/	+∞	+∞	/	/
*δ* = 0.2	13	0.8264	0.0066	0.3439	14	0.5938	0.0083	0.6726
*δ* = 0.3	12	1.0044	0.0065	0.3615	13	0.7563	0.0096	0.7478
*δ* = 0.4	11	1.1823	0.0067	0.3685	13	0.9559	0.0106	0.7459
*δ* = 0.7	11	1.9340	0.0076	0.4421	11	1.1574	0.0088	0.7205
*δ* = 1.0	11	2.7070	0.0089	0.5009	11	1.8252	0.0099	0.7045

### Validation

**Fig 8:**
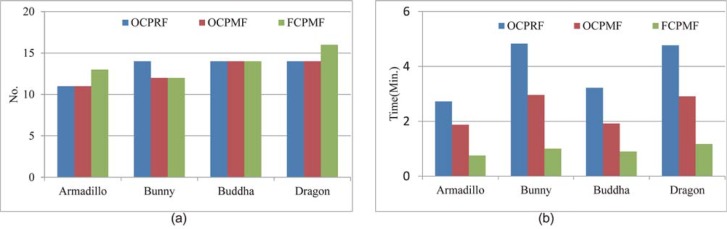
Comparison of efficiency for different approaches based on multi-scale descriptors. (a) Required number of pair-wise registrations. (b) Run time.

To validate the proposed approach, it is compared with two versions of multi-view registration approaches based on multi-scale descriptors: multi-view registration approach based on original correspondence propagation with rude fusion and multi-view registration approach based on original correspondence propagation with model fusion, which are abbreviated as OCPRF and OCPMF, respectively. As the proposed approach is based on fast correspondence propagation with model fusion, it is abbreviated as FCPMF. The performances of multi-view registration are measured by runtime and registration error. Fig 8 displays the number of pair-wise registrations and runtime required to achieve multi-view registration for each approach. Table 2 illustrates registration error of each approach tested on four datasets.

**Table 2:** Comparison of accuracy for different approaches based on multi-scale descriptors, where small value indicates accurate registration and bold number denotes the best results.

Datasets	Scan No.	OCPRF		OCPMF		FCPMF	
		*e*_R_	e_t_	*e*_R_	e_t_	*e*_R_	e_t_
Armadillo	12	**0.0068**	**0.5911**	0.0099	0.7373	0.0096	0.7478
Bunny	10	**0.0065**	**0.3356**	0.0087	0.4877	**0.0065**	0.3615
Buddha	15	0.0230	**1.3961**	0.0242	1.4553	**0.0225**	1.4977
Dragon	15	**0.0176**	**1.6343**	0.0192	1.7520	0.0190	1.7384

As shown in Fig 8, these three approaches require almost the same number of pair-wise registrations, which is a little more than the number of range scans to be aligned. As all these three approaches utilize the proposed reliability judgment, it is reasonable to conclude that this judgment is very effective for pair-wise registration. Sometimes, FCPMF may require a little more number of pair-wise registrations than other two approaches. In some scan pairs with low overlap percentages, there are a small number of reliable seed matches, which are all outside the top 30%. For these scan pair, the application of OCPRF and OCPMF may obtain good pair-wise registration results due to the application of correspondence propagation to all seed matches. Since FCPMF only applies the correspondence propagation to the top 30% sorted seed matches, it is unable to get good registration results. Besides, OCPRF may also require a little more number of pair-wise registrations than other two approaches. This is because the rude fusion results in many redundant points with multi-scale descriptors, which may reduce the robustness of pair-wise registration. Although FCPMF may require a little more number of pair-wise registrations, it is more efficient than other two approaches. By selecting the top 30% sorted seed matches, it can accelerate the correspondence propagation, which will reduce the runtime of pair-wise registration. With the model fusion, it deletes repeated range point with multi-scale descriptors so as to further improve the efficiency of subsequent registration. Therefore, both fast correspondence propagation and model fusion are helpful for the efficiency of multi-view registration.

As displayed in Table 2, all these three approaches can obtain accurate registration results, where the results of OCPRF are slightly more accurate than that of other two approaches. This is because the rude model fusion keeps many redundant range points, which is good for accurate registration. However, it leads to high computation complexity. Since many exiting approaches can refine initial registration parameters, efficiency is more important than accuracy in global multi-view registration. Accordingly, OCPRF is not a good choice. For efficient registration, the proposed model fusion is necessary to delete redundant range point with multi-scale descriptors. As most reliable seed matches contain small distances, it is easy to find good match set by the application of correspondence propagation to the top 30% sorted seed matches. Compared with raw correspondence propagation, fast correspondence propagation not only improves efficiency, but also obtains more accurate multi-view registration results.

In one word, the proposed approach is a reasonable and effective solution for the global registration of unordered range scans.

### Comparsion

To demonstrate its performance, the proposed approach was compared with two related approaches; there are automatic multi-view registration approach based on surface consistency (SurfC) [23] and automatic multi-view registration approach based on spanning tree (SpanT) [25]. Multi-view registration results are also reported in the form of runtime and registration error.

#### Accuacry and efficiency

**Fig 9:**
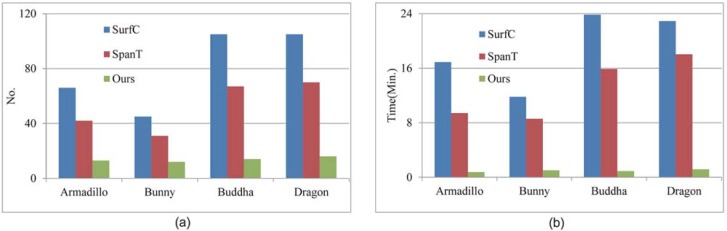
Efficiency comparison of different approaches tested on four data sets. (a) Required number of pair-wise registrations. (b) Run time.

For comparison of efficiency and accuracy, experiments were carried on four data sets. Fig 8 illustrates the number of pair-wise registrations and runtime required to achieve multi-view registration for all competed approaches. What’s more, Table 3 displays registration error of all competed approaches tested on four datasets. To demonstrate comparison in a more intuitive manner, Fig 9 displays the registration results of four data sets for all competed approaches in the form of cross-sections.

**Table 3:** Comparison of accuracy for different approaches, where small value indicates good performance and bold number denotes the best result.

Datasets	Scan No.	SurfC [23]		SpanT [25]		Ours	
		*e*_R_	e_t_	*e*_R_	e_t_	*e*_R_	e_t_
Armadillo	12	0.0195	0.8467	0.0170	1.0233	**0.0096**	**0.7478**
Bunny	10	0.0262	1.4734	0.0242	1.3588	**0.0065**	**0.3615**
Buddha	15	0.0831	1.4952	0.0851	1.2455	**0.0225**	**1.4977**
Dragon	15	0.0245	1.4285	0.0219	1.7874	**0.0190**	**1.7384**

As shown in Fig 9, SurfC requires more pair-wise registration results than other two approaches and the proposed approach requires the least number of pair-wise registrations among all these competed approaches. Actually, SurfC should apply pair-wise registration to all scan pairs involved in multi-view registration. Given *N* range scans, SurfC requires *N*(*N* − 1)/2 pair-wise registrations. Therefore, this approach is inefficient. For multi-view registration, SpanT should find at least (*N* − 1) reliable pair-wise registration of raw scan pairs to construct completed spanning tree. As most of raw scan pairs contain low overlap percentages, it also requires a large number of pair-wise registrations. While, the proposed approach only requires a small number of pair-wise registrations to achieve multi-view registration. During multi-view registration, the proposed approach utilizes the reliable pair-wise registration to augment the model shape, which can increase the overlap percentage between the model shape and data shape. With the increased overlap percentages, it becomes easily in computing reliable pair-wise registration. Hence, the number of required pair-wise registrations is reasonably reduced. Since the proposed approach utilizes the pair-wise registration by fast correspondence propagation of multi-scale descriptors, it is more efficient than other two approaches.

**Fig 10:**
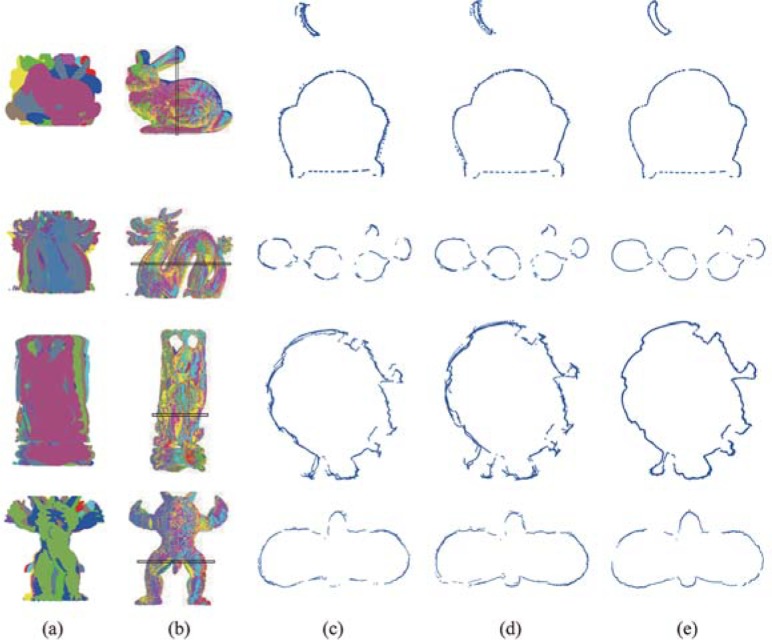
Registration results of different approaches in the form of cross-section. (a) Unordered scans (b) Reconstructed 3D models. (c) Results of SurfC. (d) Results of SpanT. (e) Our results.

As shown in Table 3 and Fig 10, the proposed approach can obtain the most accurate results for multi-view registration among all competed approaches. Since these competed approaches utilize the pair-wise registration to achieve multi-view registration, they all suffer from the problem of error accumulation. In SurfC and SpanT, the pair-wise registration algorithm is directly applied to raw scan pairs. Since few raw scan pairs have high overlap percentages, few reliable results of pair-wise registration are very accurate, which will lead to large accumulated error in multi-view registration. While, the proposed approach uses reliable pair-wise registration results to augment model shape, which can increase overlap percentages of scan pairs to be aligned. With increased overlap percentages, results of pair-wise registration become more reliable and accurate, which will reduce accumulated error in multi-view registration. Therefore, the proposed approach can always obtain the most accurate results for multi-view registration. As all completed approaches utilize results of pair-wise registration to achieve multi-view registration, the defined multi-view registration can be viewed as the mean error of pair-wise registration. As shown in Table 3, pair-wise registration error of our approach is also small than that of other approaches.

#### Robustness

For the comparison of robustness, all considered approaches were tested on Stanford Bunny with five groups of different order. Multi-view registration results are also reported in runtime and registration errors. Table 4 illustrates registration results of different approaches.

**Table 4:** Comparison of robustness for different approaches, where small value indicates good performance and bold number denotes the best result.

ID	SurfC [23]			SpanT [25]			Ours		
	*e*_R_	e_t_	T(min.)	*e*_R_	e_t_	T(min.)	*e*_R_	e_t_	T(min.)
Order1	0.0262	1.4734	11.8039	0.0242	1.3588	8.5779	**0.0065**	**0.3615**	**1.0044**
Order2	0.0280	1.2832	11.7961	0.0295	1.3697	6.0479	**0.0075**	**0.3264**	**0.9908**
Order3	0.0346	1.9781	11.4876	0.0308	1.6397	9.3768	**0.0062**	**0.4645**	**1.2782**
Order4	0.0360	2.1528	12.0357	0.0282	1.5158	9.4409	**0.0052**	**0.3472**	**1.0956**
Order5	0.0289	1.7500	11.9747	0.0296	1.3778	6.2444	**0.0078**	**0.3908**	**1.1849**

As shown in Table 4, all considered approaches are robust to the order of multiple range scans and the proposed approach can obtain the most accurate and efficient registration results for Stanford Bunny under different orders. Although the order of input range scans may be changed, all these three approaches utilize reliable pair-wise registration results to estimate rigid transformations for multi-view registration. Therefore, they can always achieve fine results for multi-view registration. As SpanT and the proposed approach only apply the pair-wise registration on some scan pairs, the change of scan order will cause their required number of pair-wise registration to be varied. Accordingly, the run time of these two approaches varies under different scan orders. However, SurfC applies pair-wise registration on all scans pairs involved in multi-view registration and its run time is relatively stable under different scan orders. By only considering the accuracy, the proposed approach is very robust.

## Application

In fact, many kinds of range scans can be viewed as the input of our approach. In this section, the proposed approach is tested on two data sets so as to demonstrate its potential applications on scene reconstruction and robot mapping.

**Fig 11:**
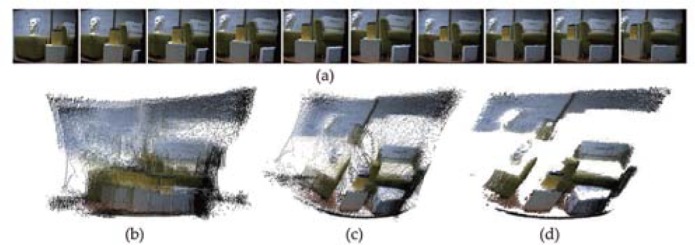
Scene reconstruction of EXBI data set. (a) Scene image of each rang scans. (b) Raw range scans. (c) Scene reconstruction with noises. (d) Scene reconstruction without noises.

For scene reconstruction, the proposed approach is tested on EXBI data set [22]. This data set contains 10 RGB-D scans recorded by the Kinect sensor, which provides range point clouds with associated color information. During the record of this data set, the Kinect sensor was manually moving in front of a indoor scene. It should be noted that only range points are utilized for scene reconstruction and color information can be used to help the final assessment. Fig 11 displays the scene reconstruction of the proposed approach. Since RGB-D contains noises, they should be filtered for good scene reconstruction. As shown in Fig 11, the proposed approach has the potential for fusing RGB-D data and can be applied to scene reconstruction.

**Fig 12:**
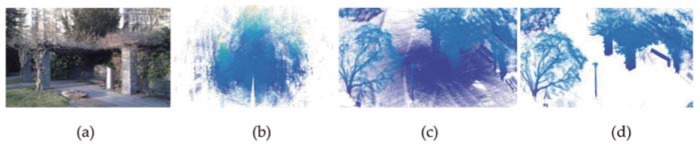
Mapping result of Gazebo data set. (a) Scene image (b) Raw range scans. (c) Mapping result with noises and ground surface. (d) Mapping result without noises and ground surface.

For robot mapping, the proposed approach is tested on Gazebo data set [28] with 32 range scans, which was obtained from a small-scale outdoor environment (72x70x19 m). During the record of this data sets, robot was equipped with a laser range finder and followed the path to form a closed loop (4x5x0.09 m). As the raw data contains range points of ground surface, they should be filtered for accurate mapping. Fig 12 displays the mapping results of the proposed approach. As shown in Fig 12, the proposed approach can achieve robot mapping with good accuracy. For small-scale environment, the proposed approach only requires a little number of reliable pair-wise registrations to achieve mapping, where the accumulation of registration error is small, results in accurate mapping. Therefore, the proposed approach can be potentially applied to robot mapping of small-scale environment. For mapping of large-scale environments, the accumulation of registration error will become very large, which may lead to the failure of mapping.

## Conclusions

This paper proposes the global approach for multi-view registration of unordered range scans. This approach is based on the pair-wise registration, which is achieved by the fast correspondence propagation of multi-scale descriptors. For multi-view registration, the reliability judgment is designed to choose reliable pair-wise registrations, which are utilized to augment the model shape. By model fusion, the overlap percentage of scan pair will be reasonably increased. With the increased overlap percentage, the pair-wise registration is able to obtain reliable accurate results, which will reduce the required number of pair-wise registration and accumulated error for multi-view registration. Experimental results conducted on public available data sets illustrate that the proposed approach can robustly achieve multi-view registration of unordered range scans with good accuracy and efficiency.

Although the proposed approach has good performance, it does not mean that this approach can solve all multi-view registration problems. Given a set of unordered scans, there may be no other scan that contains high overlap percentage to one scan. If this special scan is viewed as the reference scan, it is difficult to obtain reliable pair-wise registration for this scan, which will lead to the failure of multi-view registration. However, it should be noted that all existing approaches for multi-view registration proposed so far share this limitation as well. In the future, we will extend this approach to robot mapping of large-scale environments.
